# Sinus metastasis of lung adenocarcinoma: a case report

**DOI:** 10.3389/fmed.2023.1323222

**Published:** 2024-01-11

**Authors:** Mingyuan Xu, Qi Sun, Xin Lv, Fangjun Chen, Shu Su, Lifeng Wang

**Affiliations:** ^1^Comprehensive Cancer Center, Nanjing Drum Tower Hospital, Nanjing, Jiangsu Province, China; ^2^Department of Pathology, Nanjing Drum Tower Hospital, Nanjing, Jiangsu Province, China

**Keywords:** lung adenocarcinoma, sinus metastasis, next-generation sequencing, EGFR-TKI, immune microenvironment

## Abstract

Metastatic carcinoma of the paranasal sinuses in lung cancer is an extremely uncommon condition. We report here a 57-year-old female patient with epidermal growth factor receptor (EGFR)-positive stage IV non-small cell lung cancer (NSCLC) with multiple bone metastases. After resistance to second- and third-generation EGFR-tyrosine kinase inhibitors (TKIs), the patient presented with headache accompanied by progressively enlarging lesions of the nasal cavity on CT scan. Further endoscopic sinus neoplasmectomy confirmed sinus metastasis of lung adenocarcinoma. Although subsequent chemotherapy and immunotherapy were both administered, the disease continued to progress, and the patient passed away 21 months after diagnosis. Combined with real-time dynamic next-generation sequencing (NGS) during the different generations of EGFR-TKI treatments and dynamic tumour microenvironment analysis, we discussed the clinical manifestations of sinus metastasis and the molecular biology and tumour immune microenvironment changes after resistance to the second-and third- generation of EGFR-TKI therapy.

## Introduction

Lung cancer is a disease prone to multiple systemic metastases, common metastases including brain, bone, liver, lymph nodes, adrenal glands, thoracic cavity, and so on (1). Clinically, it is very rare for lung cancer to metastasize to unusual sites, such as the paranasal sinuses. Here, we report a patient with advanced lung cancer with EGFR-sensitive mutation who presented with recurring headaches during sequential second- and third-generation EGFR-TKI therapy, and it turned out to be sinus metastasis of lung adenocarcinoma by the following imaging and pathological examination. Unfortunately, the patient did not respond well to subsequent platinum-based doublet chemotherapy or immune therapy. Previous clinical data showed that EGFR-positive patients have a limited response to immunotherapy, and the underlying mechanism is uncertain (2, 3). Based on the patient’s multiple prebiopsy tissue samples, dynamic NGS tests and the tumour microenvironment were further analyzed. We discuss this rare metastasis of lung cancer in terms of molecular biology, tumour immune microenvironment characteristics, and possible effects of sequential treatments on the evolution of tumour biological features.

## Case presentation

**Figure 1:**
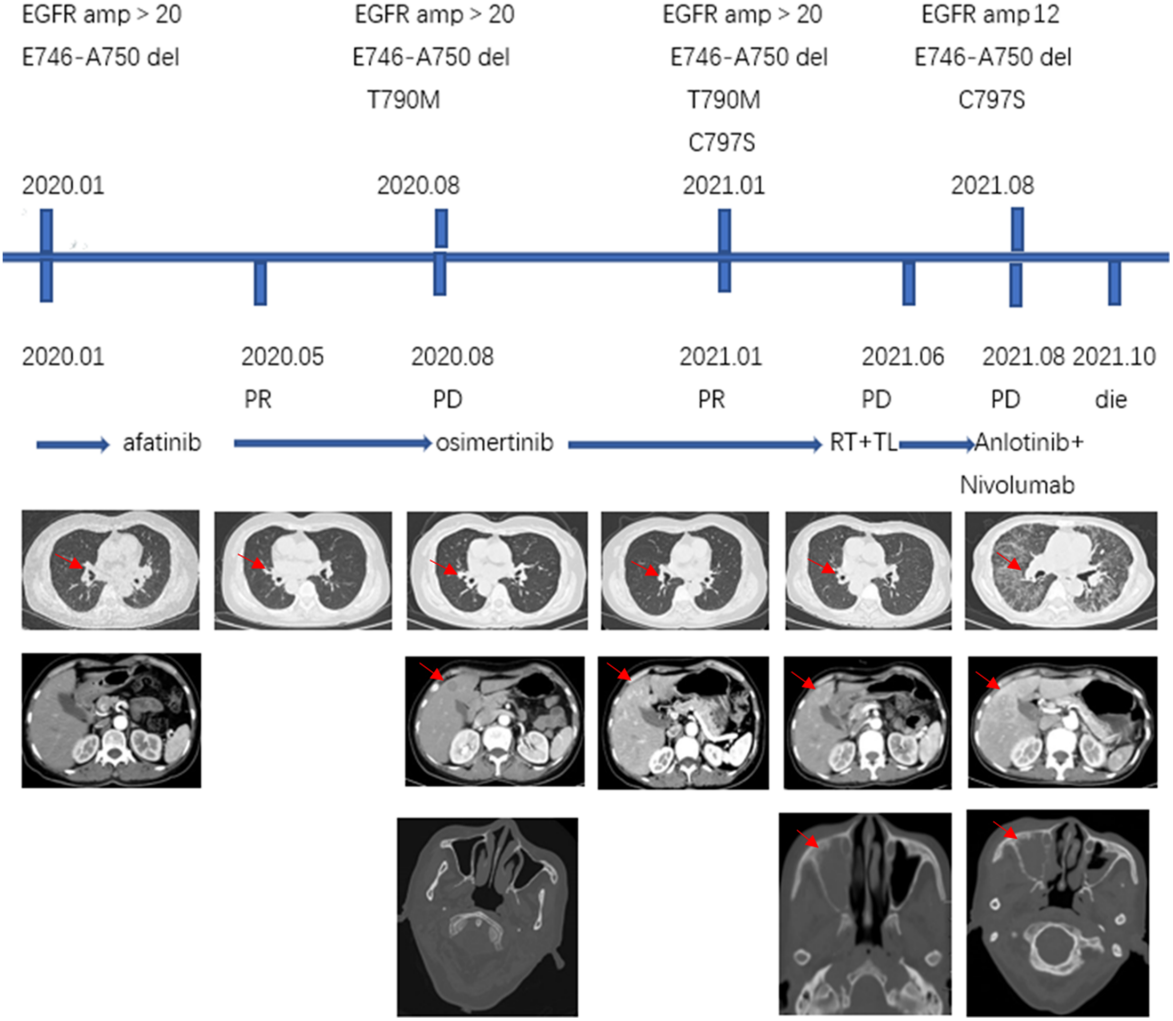
Treatment process: NGS results, treatment methods, CT (lung, abdomen, head; the red arrows on the first row of CT scans point to the lung lesions, the red arrow on the second row CT scans points to liver metastases and the red arrow on the third row CT scans points to sinus metastases). NGS, next-generation sequencing; amp, amplification; RT, radiation therapy; TL, paclitaxel liposome + lobaplatin.

A 57-year-old never-smoking female was admitted to our hospital with a paroxysmal headache complaint in January 2020. Magnetic resonance imaging (MRI) of the brain showed no obvious abnormality, and brain computed tomography (CT) showed no mass in the paranasal sinus. Enhanced CT scans of the chest revealed a left hilar mass with mediastinal and left hilar lymphadenopathy. Further transbronchial biopsy confirmed non-small cell lung cancer (NOS). Emission computed tomography (ECT) of the bone confirmed multiple bone metastases from the tumour. Due to the lack of enough tumour tissues for molecular analysis, next-generation sequencing (NGS) of blood samples showed EGFR exon 19 deletion. Hence, the patient was diagnosed with EGFR-positive stage IV non-small cell lung cancer (bone, NOS). Afatinib 40 mg daily was initiated as first-line treatment starting in February 2020, and 5 months later, it was further reduced to 30 mg due to the grade III mucocutaneous reaction. The headache was relieved after treatment, and the best response was partial response (PR) according to the Response Evaluation Criteria in Solid Tumour version 1.1 (RECIST 1.1). The disease progressed after 6 months, with repeat CT scans showing new multiple lesions in the liver. Further liver biopsy demonstrated metastatic adenocarcinoma, and PD-L1 expression on tumour cells (clone: 22C3) assessed on the basis of tumour proportion score (TPS) was <1%. NGS of liver tissues revealed the original EGFR exon 19 deletion and the exon 20 T790M mutation. Osimertinib (80 mg daily) was administered as a second-line treatment in August 2020. The patient had a PR and 7 months PFS to Osimertinib treatment. However, in April 2021, the patient complained of headache aggravation and lumbago. CT showed new liver metastasis and osteolytic lesions of the lumbar spine, and repeat ECT confirmed the progression of bone lesions. The patient then received doublet paclitaxel and platinum chemotherapy as the third-line treatment, as well as local radiotherapy of lumbar vertebra metastatic lesions (GTV 3 Gy/10 f) in April 2021. Since the lessening of headache pain, lumbar puncture was also performed with negative cerebrospinal fluid tests. In June 2021, a CT scan showed an enlarged lesion in the sinus. After ear, nose, and throat (ENT) consultation, local surgical treatment was suggested because of an unidentified sinus mass. In August 2021, nasal endoscopic sinus neoplasm resection was performed, and the postoperative pathological results demonstrated metastatic adenocarcinoma of lung origin with PD-L1 (22C3)-the negative expression of tumour cells (TPS<1%). Further NGS of sinus tissue revealed EGFR exon 20 cis-C797S missense and EGFR exon 19 deletion. In August 2021, enhanced CT showed extensive disease progression in the bilateral lungs, and anlotinib, a multitarget VEGFR TKI plus nivolumab, was administered as the fourth-line therapy. Unfortunately, the patient did not respond to the latter therapy and died in October 2021 with an overall survival (OS) of 21 months (Figure 1).

## Discussion

**Figure 2:**
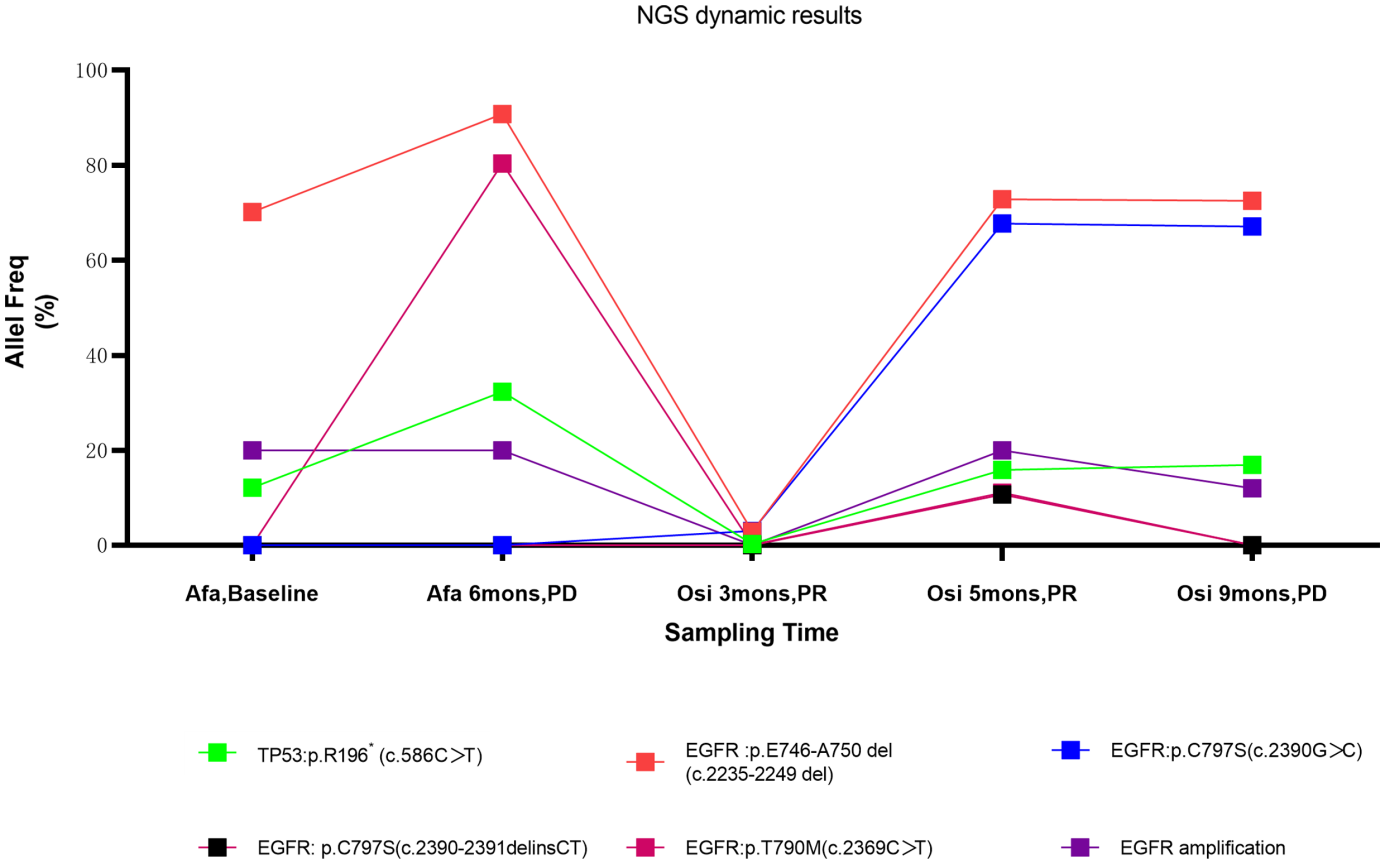
NGS changes in peripheral blood and tissue; Afa, Afatinib; Osi, Osimertinib; mons, months.

Lung cancer is the leading cause of tumours worldwide, with the highest morbidity and mortality in China (4–7). Approximately 30–50% of lung cancer patients are diagnosed with stage IV disease, and the main sites of metastasis in non-small cell lung cancer (NSCLC) include the brain (47%), bone (36%), liver (22%), adrenal gland (15%), thoracic cavity (11%), and distant lymph nodes (10%) (8). Liver (35%) and brain (47%) metastases were common in patients with metastases from SCLC, whereas bone (39%) and respiratory (22%) metastases were common in adenocarcinoma (9). However, lung cancer metastasis to the paranasal sinuses is very rare. We searched PubMed^1^ and CNKI^2^ with the keyword lung cancer and paranasal sinus metastasis from 2001 to 2021, and only 10 cases of lung cancer metastasis to the paranasal sinuses were found (Table 1). All cases were non-small cell lung cancer with adenocarcinoma (8 cases) being the most common histological type and maxillary sinus (7 cases) being the most popular involved sites of these 10 patients, headache was the main symptom in 4 patients, nasal obstruction in 3 patients, epistaxis in 2 patients, nasal swelling in 2 patients and blurred vision in 2 patients. According to our search data, male is more likely to develop sinus metastases from lung cancer and 6 patients did not mention metastases from other sites, 3 had bone metastases, and 1 had brain metastases. In our case, the patient presented with headache at the time of initial diagnosis, when CT scans showed no definite sinus mass. During the patient’s course of disease with recurrent symptoms of headache, brain or meningeal metastasis was once suspected clinically and closely observed, and finally, sinus metastasis of lung cancer was pathologically confirmed. When we retrospectively reviewed previous CT scans and found that the sinus mass progressed during sequential EGFR-TKI treatment, sinus metastasis should have been considered, especially in EGFR-positive patients with headache complaints and negative central nervous system tests. Intriguingly, the patient’s duration of response to both afatinib (PFS 6 months) and (PFS 8 months) treatment was not as long, although she harboured the EGFR common sensitive mutation, exon 19 del. The persistence of EGFR high-copy number amplification combined with TP53 mutation during multiple dynamic tissue/peripheral blood genetic tests may be one of the main reasons for poor efficacy of EGFR-TKIs treatment (20) (Figure 2). Multiple studies have confirmed that TP53 mutation is a negative prognostic factor in NSCLC patients and a negative predictor of EGFR-TKI treatment in patients with EGFR mutation (20–23). Furthermore, TP53 mutations impact the natural history of EGFR- mutant NSCLC at least partially by allowing tolerance of a greater degree of genomic instability, which results in a higher somatic mutation burden and mutagenic potential (21, 23). EGFR amplification has been shown to be a poor prognostic factor in EGFR-mutated NSCLC patients treated with EGFR TKIs (24). In addition, during treatment with osimertinib, the EGFR exon 20 cis-C797S mutation was observed in plasma ctDNA before clinical imaging progression, suggesting the potential role of liquid monitoring in EGFR TKI resistance. Niederst identified a C797S resistance mutation and determined that the position of T790M affects the efficacy of therapeutic strategies (25). If the two mutations are in trans (on different alleles), a combination of first- and third-generation TKIs can inhibit EGFR. In contrast, if the two mutations are in cis (on the identical allele), the tumor cells are refractory to any of the EGFR TKIs that they tested as well as to the combination of first- and third-generation inhibitors. In contrast, if the two mutations are in cis (on the identical allele), the tumor cells are refractory to any of the EGFR TKIs that they tested as well as to the combination of first- and third-generation inhibitors. A study provides clinical evidence that combined targeted therapy of brigatinib and cetuximab could provide benefits and may potentially be an effective treatment strategy to improve survival outcomes of patients who acquired EGFR T790M-cis-C797S-mediated resistance to Osimertinib (26). However, there is no standard of care for NSCLC patients harbouring T790M-cis-C797S. Furthermore, loss of EGFR Exon 20 T790M was found after third-generation TKI resistance. Studies have shown that with the evolution of gene clones during EGFR-TKI treatment, there is loss of the T790M mutation, which may be accompanied by other gene mutations and is more conducive to the survival of drug-resistant clones during EGFR-TKI treatment. Patients without the T790M mutation or with EGFR C797S after osimertinib resistance usually have a worse clinical prognosis (27). In our case, loss of EGFR Exon 20 T790M and harbouring C797S are the main reasons for poor prognosis.

**Table 1:** Reported cases of lung cancer with paranasal sinus metastasis, 2001–2022.

Year	Author	Histological type	Age/Sex	Involved sinus	Metastases symptoms	NGS	Outcome
2002	Zhang (10)	Squamous cell carcinoma	50/M	Paranasal	Nasal swelling epistaxis	NR	NR
2002	Clarkson (11)	Adenocarcinoma	79/F	frontal	Blurred vision Headache	NR	NR
2005	Rombaux (12)	Adenocarcinoma	71/M	frontal	Headache	NR	Survival (9mos)
2009	Chun-Ta Huang (13)	Adenocarcinoma	59/F	Paranasal	Nasal swelling	NR	Survival (2mos)
2010	Ma (14)	Adenocarcinoma	67/M	Maxillary	Epistaxis Hyposmia	NR	NR
2013	Luo (15)	Adenocarcinoma	61/M	Maxillary	Nasal obstruction Headache	NR	Survival (5 mos)
2015	Ates (16)	Adenocarcinoma	51/M	Maxillary	Blurred vision Eye pain	NR	NR
2016	Liang (17)	Adenocarcinoma	59/M	Maxillary	facial numbness Toothache	NR	NR
2016	Li (18)	Adenocarcinoma	46/F	Maxillary	Nasal obstruction	NR	NR
2021	Xu (19)	Adenocarcinoma	64/M	Sieve	Nasal obstruction Headache	EGFR mutations	Survival (13 mos)

Outcome: survival or follow-up period after presence of paranasal metastasis; F, female; M, male; NR, not reported; mos, months.

Although EGFR-mutant patients can benefit from EGFR TKIs to some extent, acquired resistance and efficient subsequent therapy are still unresolved. Recently, immunotherapy has opened a new avenue in many solid tumours, including lung cancer; however, current clinical studies have suggested that the efficacy of PD-1/PD-L1 immunotherapy is poor in NSCLC patients harbouring EGFR-sensitive mutations (2, 3). Further studies showed that the negative tumour microenvironment, including a lack of CD8+ T-cell infiltration (28), low levels of active cytokines (29), and high levels of immune-negative regulatory cells in EGFR-positive tumours, may be the underlying mechanism (30). Interestingly, the local tumour tissue microenvironment might be positively regulated by efficient EGFR TKI treatment, leading to infiltration of CD8+ T cells and an increased proportion of M1 macrophages. Thus, the exploration of the right timing of immunotherapy interventions may be the key to further improve the efficacy of immunotherapy in patients with EGFR mutations in the future (31). However, previous studies were mostly based on the use of first-generation EGFR TKIs, and the effect of third-generation EGFR TKIs on the tumour microenvironment is still unknown.

**Figure 3:**
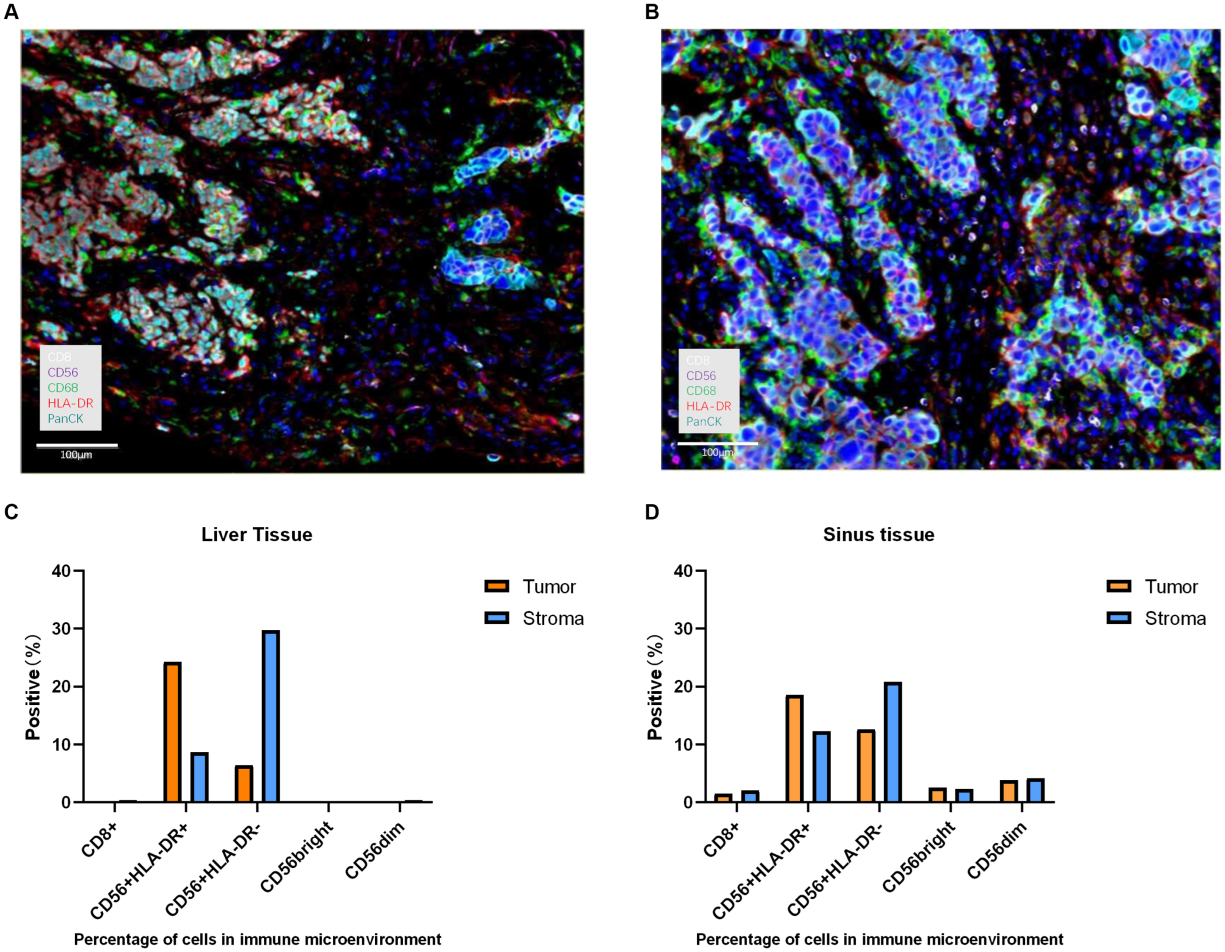
The histopathological characteristics of the tumour in the liver and sinus demonstrated by immunohistochemical staining. **(A)** expression level of the liver for CD8 (white), CD56 (purple), CD68 (green), HLA-DR (red), and Panck (cyan) was displayed in the immunohistochemical image (magnification, x100). **(B)** expression level of the sinuses for CD8 (white), CD56 (purple), CD68 (green), HLA-DR (red), and Panck (cyan) was displayed in the immunohistochemical image (magnification, x100). **(C)** Percentage of cells in the immune microenvironment of the liver. **(D)** Percentage of cells in the immune microenvironment of sinuses.

In our case, the patient did not respond to subsequent immunotherapy after resistance to osimertinib. To better understand the influence of third-generation EGFR TKIs on the TME and the possible reason for immune resistance, we found that CD8+ T-cell infiltration was consistently lacking in both the tumour parenchyma and stromal areas before and after osimertinib resistance, and the overall TME had characteristics of the immune desert type (Figure 3). Studies have shown that CD8+ T-cell density in tumour tissue has some correlation with the response and prognosis of patients to immunotherapy (32). In addition, we noticed that tumour-associated macrophages (TAMs) in the immune microenvironment of patients also changed in response to treatment with third-generation EGFR-TKIs. TAMs can be divided into the M1 type (antitumour) and M2 type (tumour-promoting) (33). The M1 type is activated by cytokines such as IFN-γ and produces proinflammatory and immunostimulatory cytokines such as IL-12 and IL-23, which are mainly involved in the inflammatory response and antitumour immune process and are related to good tumour prognosis. The M2 type, on the other hand, is activated by Th2-derived cytokines such as IL-4, IL-10, and IL-13 and mainly plays a tumour-promoting and immunosuppressive role. CD68 + HLA-DR+ cells are currently widely accepted as M1 markers and can be used to distinguish M1/2 macrophages. In this patient, the proportion of positive M1 macrophages in the TME was significantly higher than that of negative M2 macrophages after second-generation EGFR TKI resistance. The proportion of negative M2 macrophages was significantly increased after third-generation EGFR TKI resistance. These results suggest that acquired resistance to osimertinib could lead to more immunoregulative features in the TME, combined with the immune desert phenotype of this patient, which also explains the lack of response of the patient to subsequent immunotherapy after the discovery of sinus metastases.

## Conclusion

In summary, we report a rare case of sinus metastasis in NSCLC patients with EGFR-sensitive mutations. Clinically, the possibility of sinus metastasis should be considered in EGFR-positive patients with lung cancer, especially those with complaints of headache and negative central nervous system tests. We reviewed relevant literature and found that there are no characteristic clinical or radiologic features for metastatic sinus tumours; however, the diagnosis can be confirmed by histopathological examination of biopsied tumour sample. During the second-and third-generation EGFR TKIs, chemotherapy and immunotherapy treatment, we explained the reason why the second-and third-generation TKIs had difficulty maintaining long-term efficacy in this patient by real-time NGS detection based on both tumour tissues and peripheral blood and further verified the importance of peripheral blood ctDNA in prejudging early disease progression (34, 35). At the same time, we further analyzed the tumour microenvironment characteristics of patients after second-generation EGFR TKI and third-generation EGFR TKI resistance and found that the lack of CD8+ T-cell infiltration in tumour tissue and the increase in M2 macrophages after third-generation EGFR TKI resistance may be one of the main reasons for ineffective immunotherapy in patients.

## Ethics statement

The studies involving humans were approved by Medical Ethics Committee of Drum Tower Hospital. The studies were conducted in accordance with the local legislation and institutional requirements. The participants provided their written informed consent to participate in this study. Written informed consent was obtained from the individual(s) for the publication of any potentially identifiable images or data included in this article.

## Author contributions

MX: Writing – original draft, Writing – review & editing. QS: Data curation, Writing – review & editing. XL: Data curation, Writing – review & editing. FC: Writing – review & editing. SS: Writing – review & editing. LW: Writing – original draft, Writing – review & editing.

## Footnotes

^1^www.pubmed.ncbi.nlm.nih.gov

^2^www.cnki.net
